# Improved solubility and corneal permeation of PEGylated curcumin complex used for the treatment of ophthalmic bacterial infections

**DOI:** 10.1371/journal.pone.0258355

**Published:** 2022-04-07

**Authors:** Muhammad Hanif, Nabeela Ameer, Qurat-ul-Ain Ahmad, Mubashir Aziz, Khalid Mahmood, Nasreen Ramzan, Hafiz Muhammad Abdur Rahman

**Affiliations:** 1 Faculty of Pharmacy, Department of Pharmaceutics, Bahauddin Zakariya University, Multan, Pakistan; 2 Institute of Pure and Applied Biology, Bahauddin Zakariya University, Multan, Pakistan; 3 Institute of Chemical Sciences, Bahauddin Zakariya University, Multan, Pakistan; 4 Department of Pharmacy, Southern Punjab Institute of Health Sciences, Multan, Pakistan; ISF College of Pharmacy, Moga, Punjab, India, INDIA

## Abstract

Naturally occurring curcumin can be used for the treatment of corneal bacterial infections with its limitation of poor solubility. Aim of the present study was to enhance solubility and permeation of curcumin for the treatment of corneal bacterial infections. For increasing solubility, curcumin and polyethylene glycol (PEG 6000) complex (1:3) was prepared by fusion melting method. Phase solubility studies were used for the calculation of Gibbs free energy of curcumin. Central composite rotatable design (CCRD) was applied for optimization of Curcumin (CUR), PEGylated Curcumin (PEG-CUR), penetration enhancer cremophore (CR). Optimized ointments were further evaluated by mucous permeation, membrane permeability and cell toxicity studies by Transwell cell, ussing chamber and Caco-2 cells respectively. Antibacterial test was also performed by agar well diffusion method. Solubility of PEG-CUR was increased up to 93±3.2% as compared to pure curcumin and content uniformity was in the range of 95–110%. Curcumin permeation from PEG-CUR ointment was increased up to 12 folds. No toxicity of Caco-2 cells for PEG-CUR even after 24h was observed. Activity index of pure CUR, PEG-CUR ointment with or without CR against *S*. *aureus and P*. *aeruginosa* was 97±2.3, 96±1.6, 95±2.5% respectively. Ointment with solubility enhanced PEG-CUR and cremophore can be used as a promising tool for the treatment of corneal bacterial infections.

## 1. Introduction

Ophthalmic drug delivery is most challenging due to its unique physiology, biochemistry and complex internal structure which limits free entry of any external substances including drugs [1]. The problem can be resolved by designing novel drug delivery systems with enhanced drug targeting ability, prolong drug residence time at ocular surface and reduction of administration frequency. The corneal epithelium is the most lipophilic portion of the cornea and tight junctions on epithelial surface prevents direct penetration of drug molecules to the deeper layers if the junctions remain intact. In recent years much attention has been focused on enhancing solubility and penetration of drug substances to improve bioavailability of poorly water-soluble drugs Different corneal penetration enhancers and hydrophilic polymers like Polyethylene glycol 400 (PEG 400), PEG 6000, PEG 600, Cremophore EL, Polyoxyethylene glycol oleyl were used according to specifications of USP. These carriers have ability to safeguard the encapsulated substances and help in the transportation to various compartments in the interior of eye [2].

Curcumin has antimicrobial, antioxidant, anti-inflammatory, anticancer activity and blocks microbial growth of *S*. *aureus and P*.*aeruginosa* [3]. It has been reported that curcumin has also beneficial effects on several ocular diseases, such as bacterial infections, chronic anterior uveitis, diabetic retinopathy and glaucoma [4]. Despite its strong therapeutic effects in ophthalmology several key challenges have limited its clinical applications including its poor solubility (~11 ng/mL) in aqueous solutions [5]. Taking into consideration the above mentioned reasons it would be advantageous to design a formulation which enhanced solubility and permeation of curcumin [6]. PEG 6000 being biocompatible, biodegradable, stable, non-toxic and water-soluble polymer with bacteriostatic properties, widely used polymer in preparing solid dispersions for increasing solubility. In this study it was used to formulate physical complex with curcumin because of its very high affinity towards water and low toxicity. In recent years, ophthalmic ointment was emerged as promising platform in ocular drug delivery increasing bioavailability by enhancing the corneal permeability or by increasing the retention time of the formulation in the ocular surface [7].

Keeping in view the above factors, our aim of study was to improve solubility of curcumin by using polyethylene glycol and enhance corneal permeation by using permeation enhancer in ointment. Composition and evaluation of ophthalmic ointment was examined using solubility studies, content uniformity, ATR-FTIR and cell permeation studies. *Ex-vivo* studies such as mucous permeation, membrane permeability was conducted. Furthermore, to study the effectiveness of more soluble more permeable curcumin, antibacterial tests of optimized ointment formulations was performed against multi drug resistant pathogens *“Staphylococcus aureus”* and “*Pseudomonas aeruginosa”*.

## 2. Materials and methods

### 2.1. Materials

Curcumin was purchased from Acros Organics, USA, M.W≈368.38g/mol. PEG 6000, white soft paraffin (M.W≈348g/mol), Triton×®100(M.W≈625g/mol), sodium pyruvate, L-glutamine and 10% fetal bovine serum (FBS) were purchased from Sigma Aldrich, Hamburg, Germany. Liquid white soft paraffin, mineral oil (M.W less than 500g/mol), cremophore EL (M.W≈136.15g/mol), acetone (M.W≈58.08g/mol), potassium dihydrogen phosphate (M.W≈136.086g/ml), sodium hydroxide (M.W≈39.997 g/mol) and Muller Hinton (MH) agar were purchased from Merck Darmstadt, Germany. Sodium chloride (M.W≈58.44g/mol), sodium bicarbonate (NaHCO_3_, MH≈84.007g/mol) were purchased from Duksan Pure Chemicals, Gyunggido, Korea. Calcium dichloride dehydrate (CaCl_2_-2H_2_0 M.W≈147.0g/mol), Hank’s balanced salt solution (HBBS) and Dulbecco’s Modified Eagle medium (DMEM) were purchased from Atlanta biologicals, Norcross, Georgia. All chemicals used in study were of analytical grade.

### 2.2. Phase solubility diagram

Phase solubility studies of CUR were performed as already described method of Hoshikawa *et al*., [8]. Briefly; aqueous solutions of increasing concentration of polyethylene glycol (PEG) 6000 (1, 2, 3, 4, 5, 10, 12%m/v) were prepared and excess amount of CUR was added in each solution. All resulted solutions were placed in water bath at 37°C for 24 h, filtered through Whatman filter paper (0.45um) and analyzed at 425nm by UV-spectrophotometer (Perkin Elmer 1900-BMS). Concentration of CUR in all above mentioned PEG solutions was calculated by using already prepared standard curve of curcumin. Gibbs free energy was a thermodynamic function calculated by using equation. Experiment was performed in triplicate to obtain mean±SD.

$${\Delta \text{G}}_{\text{o}}=-2.303{\text{RTlogS}}_{\text{o}}/{\text{S}}_{\text{s}}$$ (1)

Where G = ideal gas constant, T = absolute temperature in Kelvin, S_o_ = solubility of CUR in different concentrations of PEG 6000, S_s_ = solubility of CUR in water (Blank).

Stability or binding constant (Ks) was calculated by using following equation [9] where S_o_ is the solubility of curcumin in absence of PEG.


$$\text{Ks}=\frac{\text{Slope}}{{\text{S}}_{\text{o}}}\left(1-\text{slope}\right)$$ (2)

### 2.3. Preparation of PEGylated curcumin complex and solubility studies

**Fig 1:**
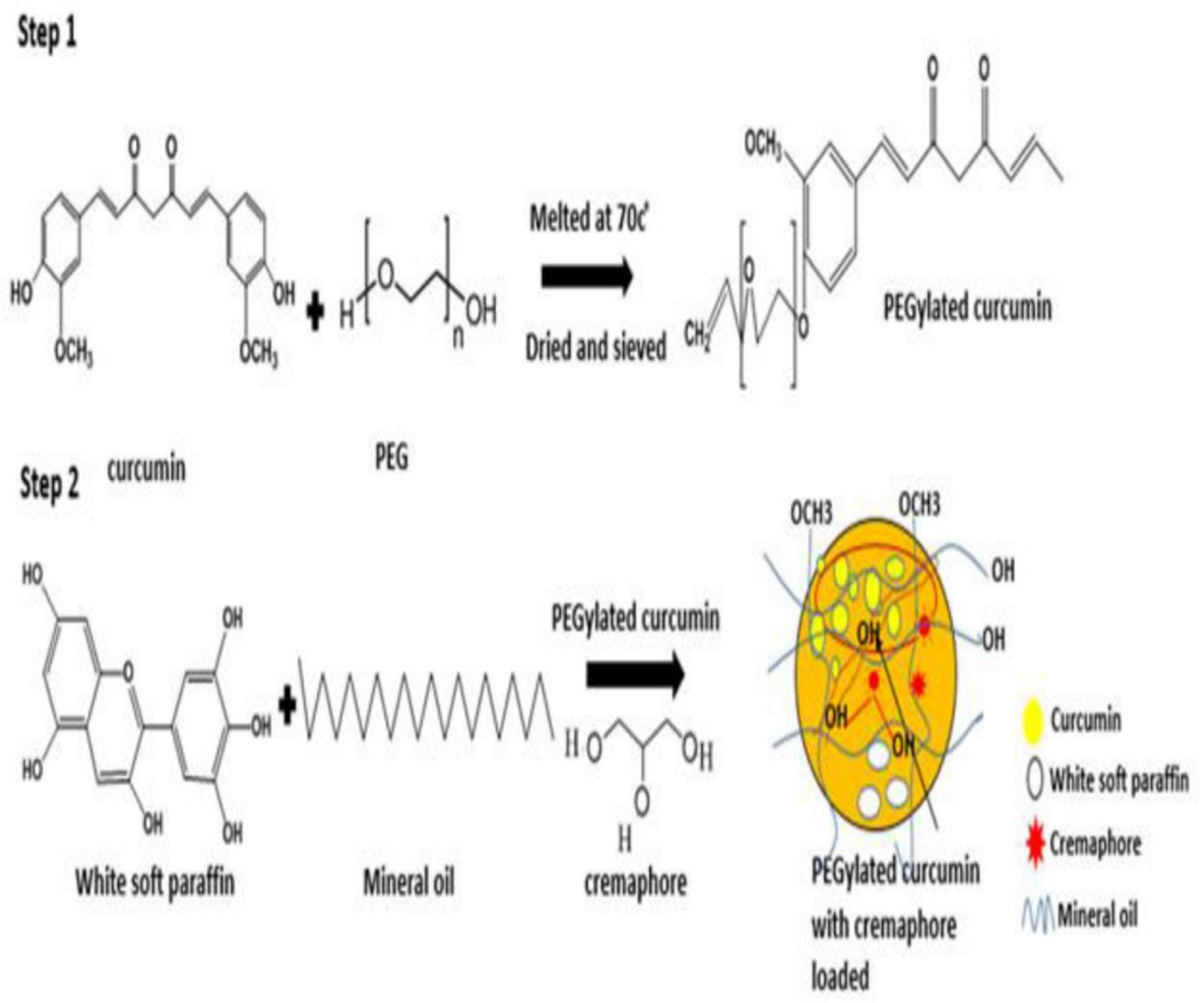
Chemical scheme of synthetic pathways for synthesis of PEG-CUR (Step 1), formulation of PEG-CUR loaded ointment with Cremophore (Step 2).

To increase the dissolution rate and solubility of curcumin, PEGylated curcumin complex (PEG-CUR) was prepared by fusion method as already reported by Nguyen *et al*., [10]. Briefly; on the basis of results of phase solubility diagram polyethylene glycol (PEG) 6000 (3.75g) was melted in a water bath at 70°C and CUR (1.25g) was dispersed into molten mixture with continuous mechanical stirring until uniform. The resulted mixture was placed in refrigerator for 4–5 min to cool, solidify and dried at room temperature. Resulted PEG-CUR complex was grinded in pestle mortar, screened from sieve (mesh #40), preserved in aluminum foil, and kept in a dry place. Moreover, confirmation of PEG-CUR complex was done by FTIR spectra as shown in Fig 4 and solubility studies. For solubility analysis through shake flask method about 20mg prepared PEG-CUR complex was dispersed in 10 ml of distilled water at 37°C at 50 rpm for 24h. After shaking for 24h samples were filtered and analyzed at 425 nm using UV-visible spectrophotometer (Shimadzu UV-1650, Tokyo, Japan) (Fig 1).

### 2.4. Central composite rotatable design

A central composite rotatable design (CCRD) was applied with three variables curcumin, PEG-curcumin, cremophore (CR) and responses were percentage permeation, solubility, and antibacterial effect. Twelve different formulations of ointments containing pure curcumin and PEG-Curcumin with quadratic model was applied. Where β° was the intercept, demonstrating the mathematical mean of all numerical outcome of 12 trials, β_1_ and β_2_ were the coefficient, which were calculated from the observed experimental values of Y and X_1_ and X_2_ were coded level of independent variables. Analysis of variance (ANOVA) was used to validate the experimental outcomes, which was described in the form of polynomial equation. Various combinations were tried to find out optimized formulation in the form of more soluble and more permeable curcumin ointment for improved antibacterial effect.


$$\text{Y}=\beta {}^{\circ}+\beta_{1}{\text{X}}_{1}+\beta_{2}{\text{X}}_{2}+\beta_{12}{\text{X}}_{1}{\text{X}}_{2}-\beta_{12}{\text{X}}_{12}-\beta_{22}{\text{X}}_{22}$$ (3)

### 2.5. Preparation of PEG-CUR ophthalmic ointment

Ophthalmic ointment formulations of CUR and PEG-CUR were prepared by fusion method as already reported by Xiaoming Xu *et al*., [9]. Briefly; out of twelve formulations, six CUR ointments were prepared by using ointment bases such as mineral oil (2.54 w/v) and white soft paraffin (6.44 w/v). The resulted mixture was melted at 70°C in a water bath and calculated amount of pure CUR was poured into molten mixture under continuous agitation of 900 rpm for 2h until uniformly blended. Cremphore was added as permeation enhancer in three of the six CUR ointment formulations. Resulted ointments (CUR-1, CUR-2, CUR-3, CUR-C4, CUR-C5, CUR C6) was permitted to cool, packed in aluminum tubes, and stored at 25±0.5°C. Same procedure was repeated for preparation of next six PEG-CUR ointment formulations with and without adding cremophore namely PEG-CUR1, PEG-CUR2, PEG-CUR3, PEG-CUR-C4, PEG-CUR-C5, PEG-CUR-C6.

### 2.6. Characterization of ophthalmic ointments

To inspect physical appearance of ointment, aluminum tubes were cut vertically into three parts. Any change in appearance or texture of ointment was observed [9]. Weighed amount of each ointment (approximately 50mg) was taken from different portions of tube and placed in a beaker containing 400 ml of phosphate buffer solution (PBS pH 7.4) and homogenized at 900 rpm for 20 min. Uniformity of CUR and PEG-CUR content dispersed in ointment was examined by UV spectrophotometer (UV 1900 BMS) at 425nm [11]. pH of each formulation was also measured with digital pH meter [12]. All the experiments were repeated in triplicate mean±SD (n = 3). FTIR (Bruker USA) spectroscopy method was performed for determining the compatibility and possible interactions between curcumin and excipients used in preparation of ointment. Spectra was recorded in the range of 4000 to 400cm^-1^ and 25 spectra were used [13].

#### 2.6.1. *In vitro* mucous permeation studies

Permeation studies of CUR and PEG-CUR through mucous was conducted as previously described by Yousef *et al*., [14] after obtaining the approval from the faculty ethical review board vide letter no 521/pharm/2021. Briefly; Freshly isolated mucus from rabbit’s eye and already prepared NaCl (0.1M, 5ml) solution were mixed (1:5) and stirred at 3–4°C for 1h. Mixture was centrifuged at 1000 rpm for 2 h at 8–10°C. The bottom layer was separated, stirred for 1h by adding 2.5 ml NaCl (0.1M) solution, centrifuged for 2h and collected bottom layer. Mucus was divided into aliquots (0.5 g) and kept at -20°C prior to experimentation. For mucus permeation study, 24 well plates contain Transwell diffusion system (Greiner-Bio-One) with 3μm pore polyester membrane (PET). Approximately 200μL already purified and defrosted mucous was placed in donor chamber and 500μL PBS (pH 7.4) was put in the acceptor chamber. 250μg of CUR-3 ointment diluted with PBS (weight ratio of 1:100) was applied in mucous donor compartment. Plates were placed on a shaking water bath at 300 rpm. At defined time intervals 100 μl of samples were withdrawn from acceptor compartment, replaced with equal quantity of PBS (pH 7.4) and analyzed by UV spectrophotometer at 425 nm. Same procedure was repeated for permeation of PEG-CUR3 and PEG-CUR-C6 ointments.

#### 2.6.2. *In vitro* drug diffusion studies

Ussing chamber was used to investigate permeation of ointment containing CUR and PEG- CUR through fresh corneal membrane of rabbit eye by using already reported method of Polat *et al*., [15]. Briefly; from rabbit eye, cornea was separated (transparent white part of eye covered the anterior chamber), washed with 100mM PBS (pH 7.4). Corneal membrane was fixed between donor and acceptor compartment of ussing chamber. 0.5g CUR-3 ointment and 1ml PBS (pH = 7.4) were placed over corneal membrane in donor chamber kept in water bath 37°C. Samples were withdrawn at predetermined time intervals and analyzed thrice by UV spectrophotometer (UV 1900 BMS) at 425nm. Same procedure was repeated for analysis of membrane diffusion or permeation of curcumin form PEG-CUR3 and PEG-CUR-C6 ointments.

#### 2.6.3. Cell viability studies

Cell toxicity assay was performed on as previously described by Patrica Severino *et al*., [16]. Briefly; 24 wells plate having 25,000 Caco-2 cells cultivated for 2 weeks within defined conditions of 5% CO_2_ at 37°C for 14 days at expected conditions of 95% humidity. Fresh MEM was used and replaced after every 48 h with 25 mM HBS (pH 7.4). White (MEM) was replaced with freshly prepared suspension (0.5 and 1% each) of Curcumin, CUR-C6, PEG-Curcumin, PEG-CUR-C6. For every 3 and 24h ointment containing well/cells were incubated at controlled conditions of humidity and CO_2_. For positive control fresh MEM and for negative control 2% Triton X-100 was used. 250μl solution of 2.2mM resazurin was added in fresh buffer containing already washed cells and incubated for 3h at 37°C [17]. Fluorescence was calculated by drawing out 100 μl sample from every well and metabolism of resazurin in a Caco-2 cell was calculated at 425nm. Same procedure was adopted for samples of 24h incubation. Following formula was used for the calculation of toxicity of Caco-2 cell [18].


$$\text{Cell}\;\text{viability}=\frac{\text{Absorbance}\;\text{of}\;\text{Sample}}{\text{Absorbance}\;\text{of}\;\text{standard}}\times 100$$ (4)

### 2.7. Antibacterial susceptibility test

To compare effectiveness of pure curcumin and more soluble and highly permeable pegylated curcumin ointment, an i*n-vitro* antimicrobial susceptibility test was performed as previously described by Sandle *et al*., [19]. Briefly; bacterial cultures of *S*. *aureus and P*. *aeruginosa* were grown in nutrient broth for 24h at 37°C. Muller Hinton (MH) agar plates were prepared and 100μl from each bacterial suspension (10^6^ cells/ml) was spread on separate plates with sterile cotton swab. In MH agar plates, wells were prepared (6 mm diameter) with aseptic borer. Samples of pure CUR, PEG-CUR, CUR-3, CUR-C6, PEG-CUR3, PEG-CUR-C6 (100 mg each) were directly applied in wells of both microbial suspension containing plates. Ciprofloxacin 5 mg/100μl was used as positive control and PBS (pH 7.4) as negative control. All plates were incubated at 37°C for 24h. Zone of inhibition (ZOI) of each sample was measured on next day. Experiment was performed in triplicate as mean±SD. Bacterial ZOI was compared with ciprofloxacin (positive control) according to CLSI guidelines 2016 and percentage zone of inhibition was calculated. The observed diameters of the zones of inhibition were measured by using formula.


Activityindex=Inhibitionzoneofsample/inhibtionzoneofstandard (5)

### 2.8. Statistical analysis

Data were expressed as mean±standard error (n = 3) and one-way ANOVA was applied to determine the significance of the p values<0.05.

## 3. Results and discussion

**Fig 2:**
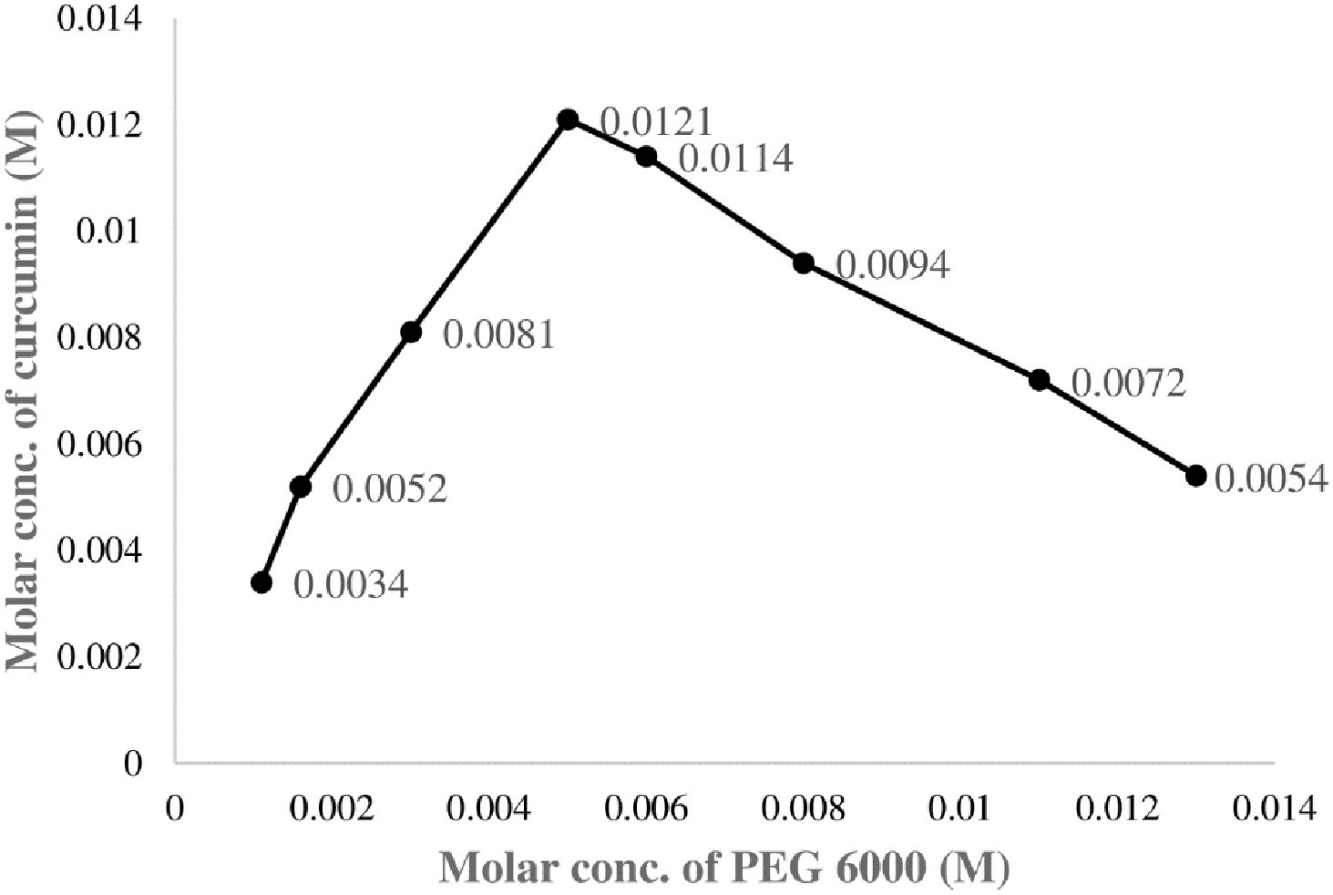
Phase solubility diagram of CUR in different molar concentrations of PEG 6000 and molar concentration of CUR showed that 3:1 PEG-CUR complex has maximum solubility of CUR represented as mean ± SD (n = 3).

The most dramatic increase in solubility of curcumin was obtained more than 20 times higher by solidly dispersed complex of CUR with PEG 6000 (1:3) than its intrinsic solubility. Following the classification of Higuchi and Connors, CUR in different PEG 6000 solutions showed A_N_ diagram. Phase solubility diagram Fig 2 showed the ratio of concentrations of PEG and CUR for making of PEG-CUR complex. Interestingly results indicated the increase in solubility of CUR from 0.0016M to 0.005M of PEG 6000 and further addition of PEG did not enhance solubility as reported by Fouad *et al*., [20]. It is clearly observed that phase solubility diagram of CUR in the presence of PEG can be classified as A_N_-type which represents that complex has linear increase in solubility up to certain limit resulting in the formation of PEG-CUR (3:1) complex. This increase in solubility might be attributed to improved wetting of CUR in the presence of PEG thus higher probability of solution formation during the formulation of solidly dispersed CUR [21].

Gibbs free energy (ΔG_0_) is a thermodynamic function and as ΔG_0_ becomes more negative, the reaction becomes more favorable and spontaneous. In the present study, obtained ΔG_0_ values become more negative with increasing PEG concentration but up to certain limit. It has been reported that the driving force for complex formation between PEG and CUR may include van der waals interaction, hydrogen bonding, and hydrophobic interaction, resulting in the formation of solution. Negative Gibbs free energy values at different concentrations of PEG 6000 polymer showed weak bonding between CUR and PEG which distinctly proved the spontaneous process of CUR solubilization at 37°C as shown in Table 1. This indicates that curcumin in different PEG solutions has more favorable environment in water and can be used for treating ophthalmic bacterial infections. Stability constant (Ks) value between 200 and 5,000 M^−1^ is considered as most suitable for the improvement of solubility and stability of poorly soluble drugs. The Ks values of PEG-CUR complex was found 484.12±2.01 M^−1^ which is within the given range. Thus, it may be concluded that the binary complexes with polymers like PEG can improve the solubility and stability of curcumin. Colour of pure curcumin changes from yellow to brownish yellow after making complex with PEG provided a clear indication of physical interaction between CUR and PEG. The solubility of pure curcumin in PBS (pH 7.4) at 37°C was very low 0.432μg/ml whereas solidly dispersed PEG-CUR complex prepared by fusion method has 25.3 μg/ml solubility which was 98% more as compared to pure curcumin. The increase in solubility might be attributed to formation of soluble physical complex of curcumin and PEG as PEG 6000 had a capability of increasing curcumin solubility.

**Table 1:** Calculation of Gibbs free energy of curcumin.

Solubility of CUR in water Blank (Ss) mg/ml	Solubility of CUR in different conc. of PEG 6000 (S_0_) mg/ml	PEG/Blank S_0_ /Ss	Log PEG/Blank Log S_0_/Ss	*ΔG(Gibbs free energy)(KJ/mol)*
0.015	0.143	9.53	0.979	-5.81
0.015	0.149	9.93	0.996	-5.91
0.015	0.499	33.2	1.521	-0.02
0.015	0.310	20.6	1.313	-7.79
0.015	0.200	13.3	1.123	-6.66
0.015	0.229	15.2	1.181	-7.00

**Fig 3:**
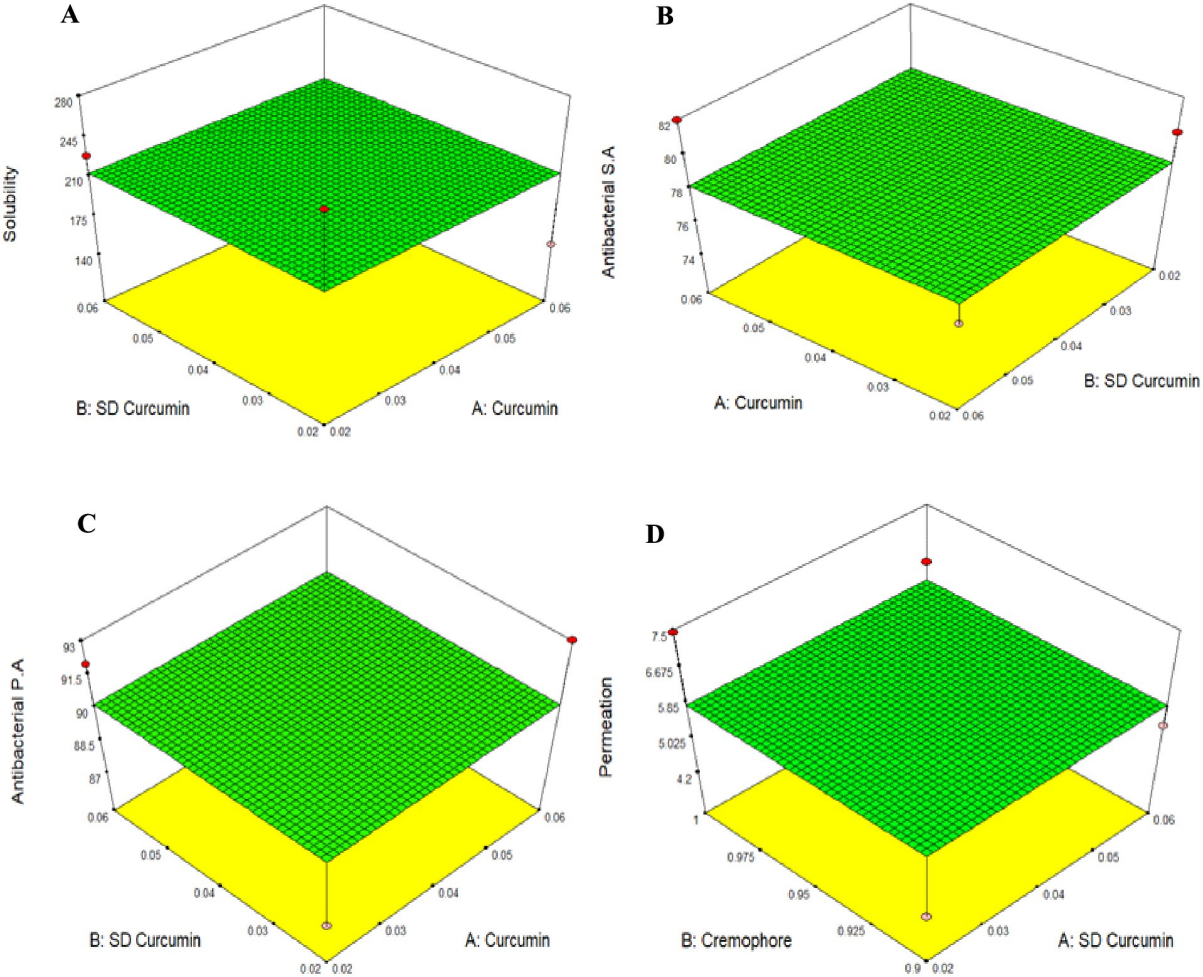
Comparative evaluation of effect of CUR and PEG-CUR (A) on solubility (B, C) antibacterial effect and (D) Cremophore as permeation enhancer for selection of ointment formulations.

Central composite rotatable design provides the rotatable 3D plots. These plots help to visualize response surfaces for preparation of ointment formulations (Fig 3). Optimum value was reached via the numerical optimization function. Total twelve formulations were prepared as shown in Table 2. Based on the results of solubility, permeation, and anti-bacterial effect four ointment formulations naming CUR-3, PEG-CUR3, CUR-C6, PEG-CUR-C6 were selected for further evaluation.

**Table 2:** Preparation of CUR and CUR-PEG ointments with oleaginous bases mineral oil (2.54 w/v) and white soft paraffin (6.44 w/v) in each formulation.

Ointment base (oleaginous)	Curcumin (w/w)	PEG Curcumin (w/w)	Cremophore (ml)
CUR-1	0.02	-	-
CUR-2	0.04	-	-
CUR-3	0.06	-	-
CUR-C4	0.02	-	1.00
CUR-C5	0.04	-	1.00
CUR-C6	0.06	-	1.00
PEG-CUR1	-	0.02	-
PEG-CUR2	-	0.04	-
PEG-CUR3	-	0.06	-
PEG-CUR-C4	-	0.02	1.00
PEG-CUR-C5	-	0.04	1.00
PEG-CUR-C6	-	0.06	1.00

**Fig 4:**
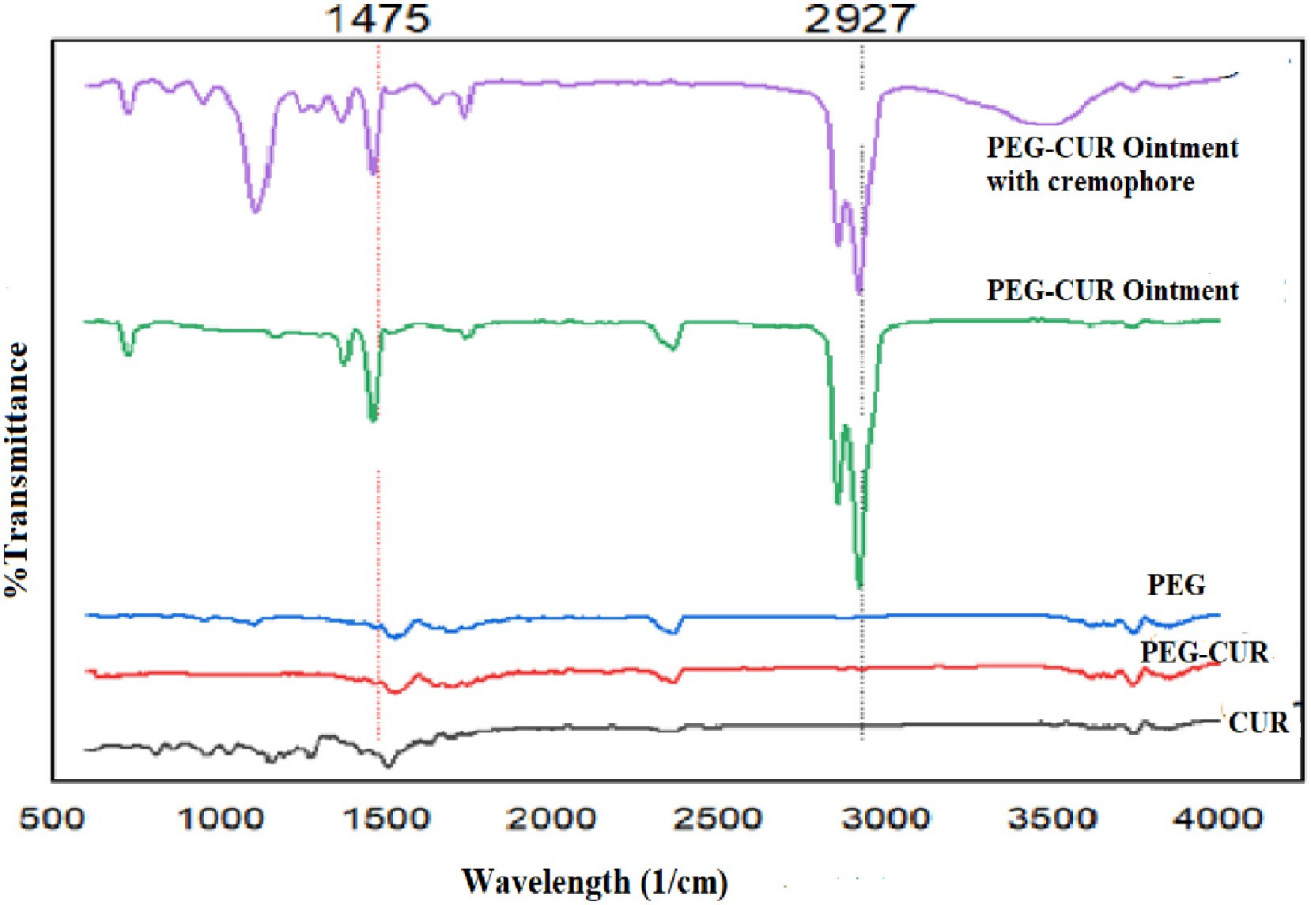
FTIR-spectra of pure curcumin, PEG-Curcumin, PEG, PEG-CUR Ointment and PEG-CUR Ointment with cremophore.

Content uniformity of all optimized formulations were found to be within the range of 95–110% which showed that CUR and PEG-CUR was uniformly distributed in ointments as previously reported by Yin, X. *et al*., [22]. Cremophore (CR) was finally selected as a penetration enhancer due to its hydrophilicity and used in ophthalmic ointment as already reported by Aboali *et al*., [23]. PEG-CUR ointment with cremophore showed enhance solubility and permeation. Results showed that CUR-3, PEG-CUR3 have pH value 5.3 and CUR-C6, PEG-CUR-C6 have 5.8 pH. The FT-IR spectra results showed in Fig 4 was characterized by strong OH bond stretching vibrations at 3506cm^1^ as previously reported by Zhang *et al*., [24]. Furthermore, C = C alkene stretching bond present at 1635cm^-1^ and C-O vibration at 456cm^-1.^. Spectrum of PEG-CUR shows broad peak at 3617cm^-1^ which indicate the presence of OH functional group as previously described by Buong Singh, P. *et al*., [25]. C-H alkene stretching was at 1750cm^-1^, strong C-O stretching was at 456cm^-1^. Spectrum of PEG 6000 shows broad peak at 3617cm^-1^ which indicate the presence of OH functional group as previously described by Gangurde, A.B., *et al* [26]. FT-IR spectra of PEG-CUR3 and PEG-CUR-C6 showed medium C-H stretching vibration at 2921cm^-1^ and 2923cm^-1^ and C-O strong bonding stretching at 1456cm^-1^ and 1150cm^-1^ respectively.

**Fig 5:**
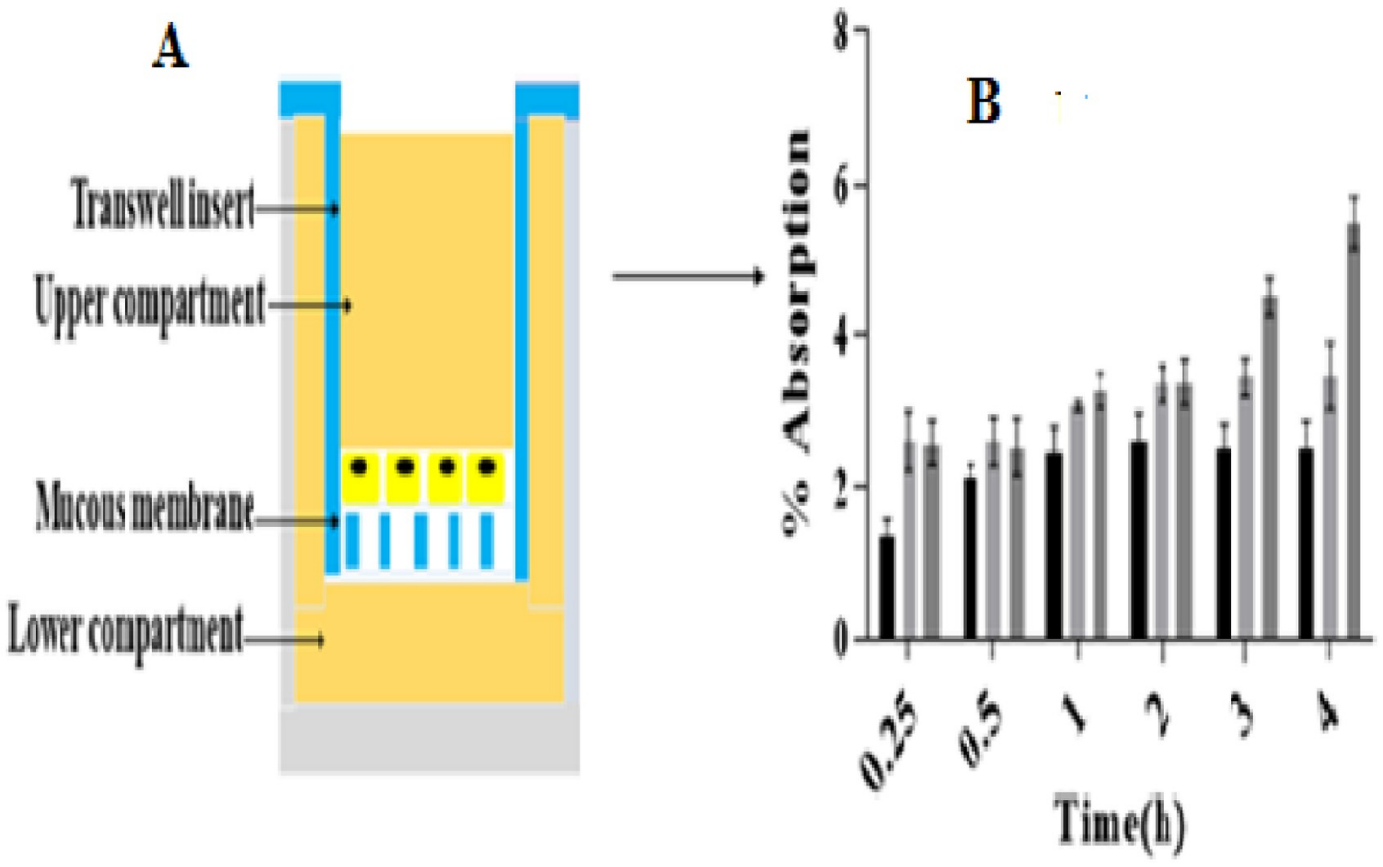
CUR absorption studies from ophthalmic mucous by using Transwell cells (A) and comparative ex-vivo mucous permeation of CUR-3 (■), PEG-CUR3 (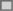) PEG-CUR-C6 (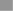) ointment formulations via transwell plates at 150 rpm after 4 h of experiment. Data is shown as mean ±SD (n = 3).

% Absorption or mucous permeability studies via Transwell cell was performed to compare transport efficiency of PEG-CUR ophthalmic ointment. Fig 5 shows comparative results of three formulations (CUR-3, PEG-CUR3, PEG-CUR-C6). It is observed that PEG-CUR-C6 3-fold more permeation of Curcumin through aqueous humour as compared to CUR-3 ointment at 4h of experiment but the rate of penetration of Cur across mucus was slow compared to corneal membrane epithelium. The low rate of Cur permeation may be due to viscous nature of aqueous humour that hinders the permeation and causes the retention of Cur in mucus. As the purpose of this study was to ensure the penetration of PEG-CUR-C6 ointment in aqueous humour so we can also use this ointment to target posterior segment of eye to treat ophthalmic diseases [27].

**Fig 6:**
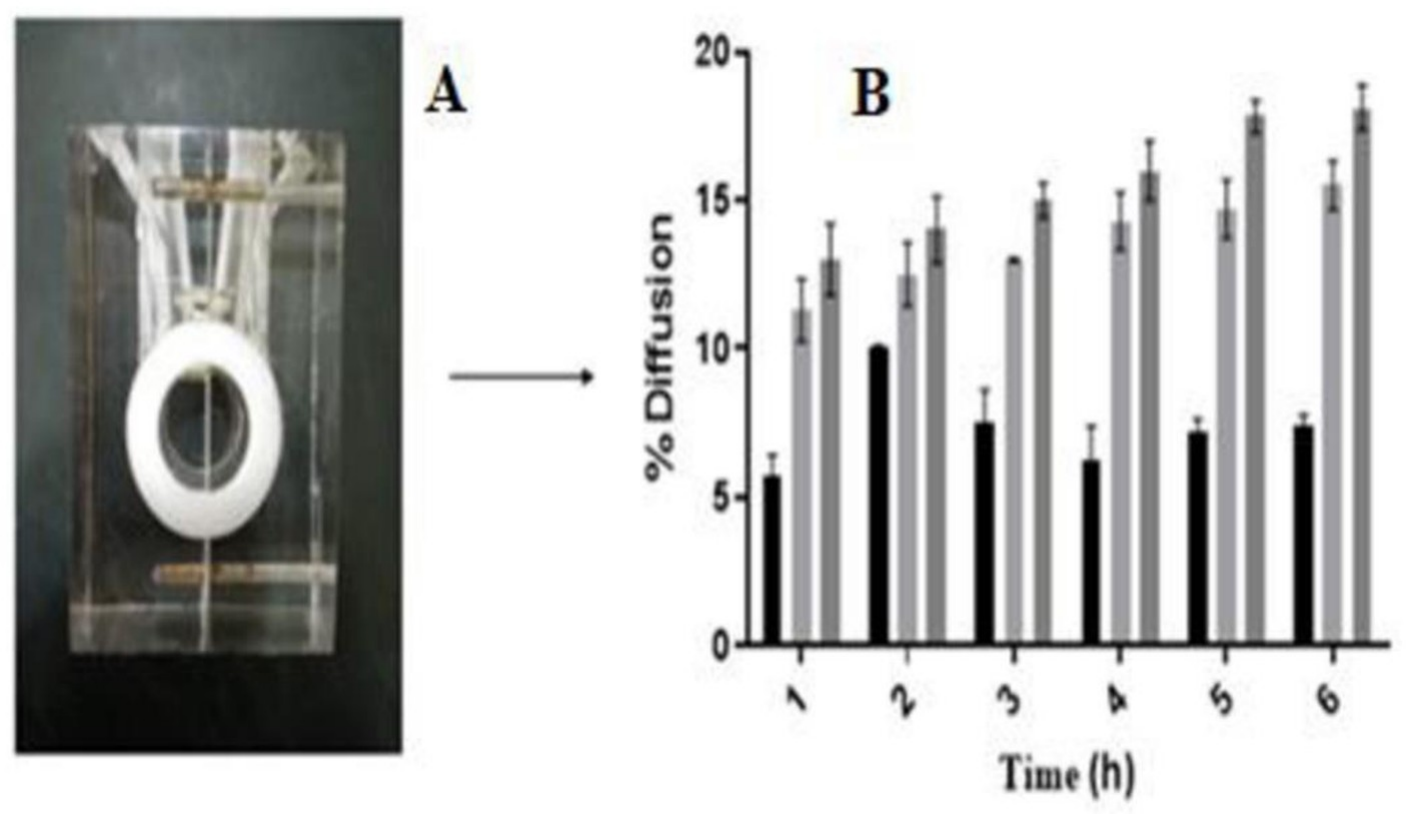
Drug diffusion study of CUR-3 (■), PEG-CUR3 (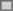) PEG-CUR-C6 (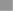) ointment formulations via ussing cell (A) at 37°C. Graph in (B) showed increase in percentage permeability of PEG-CUR-C6 as compared to the Cur and PEG-CUR ointments across corneal membrane. Data is shown as mean ±SD (n = 3).

Membrane permeation or drug diffusion studies via ussing chamber of CUR-3, PEG-CUR3 and PEG-CUR-C6 ointment formulations was performed through corneal membrane layer as previously reported by Adele Farall *et al*., [28]. Fig 6 showed comparative *ex-vivo* Trans corneal diffusion percentages of optimized formulations in which PEG-CUR3 increased percentage diffusion 6.5-fold and PEG-CUR-C6 showed maximum percentage diffusion up to 12-fold compared to the CUR-3 (*p*<0.05). The higher permeability of curcumin from PEG-CUR-C6 could be attributed to the presence of solidly dispersed PEG-CUR complex and cremophore as permeation enhancer. Only dissolved drug molecules are able to permeate corneal membrane so PEG-CUR complex enhanced the solubility of curcumin and cremopore additionally enhance permeation as previously reported by Moiseev *et al*., [29].

Cell viability test was performed on Caco-2 cells exposed to 0.5% and 1% (w/v) suspension of pure Curcumin, CUR-C6, PEG-Curcumin, PEG-CUR-C6. Cell viability was assayed with Resozurin indicator, which after exposure to active cells converts from dark blue color (dead cells) to green color (alive cells). Positive control (MEM) showed 100% cell viability [30].

**Fig 7:**
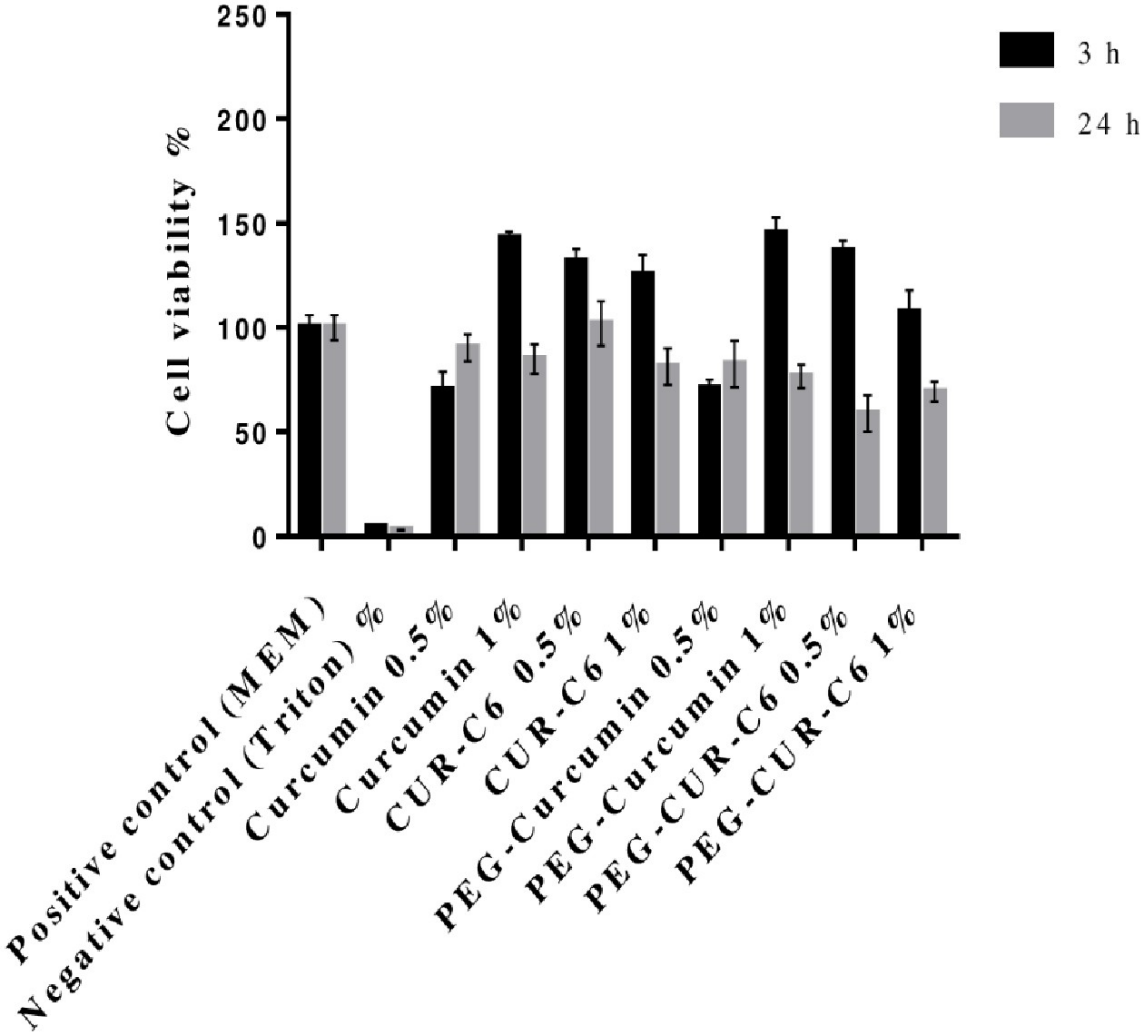
Represents Caco-2 cell viability after 3h (black bar) and 24 h (grey bar) of incubation at 37°C of pure Curcumin, CUR-C6, PEG-Curcumin, PEG-CUR-C6 (0.5% and 1% w/v each) as compared to 2% triton X-100 (negative control) and MEM (positive control). Data is shown as mean±SD (n = 3).

Fig 7 shows that Caco-2 cells can excellently withstand with formulation as no cell toxicity noticed for up to 3h with 0.5% and 1% suspension (w/v). While continuous exposure of Caco-2 cells with formulations for 24 h resulted in average reduction of 52% of cell toxicity. Results concluded that this formulation is toxic for Caco-2 cells indicating concentration dependent toxic effect of CUR as previously reported by Patrica Servino *et al*., [16] and no toxicity was observed against normal cells.

**Fig 8:**
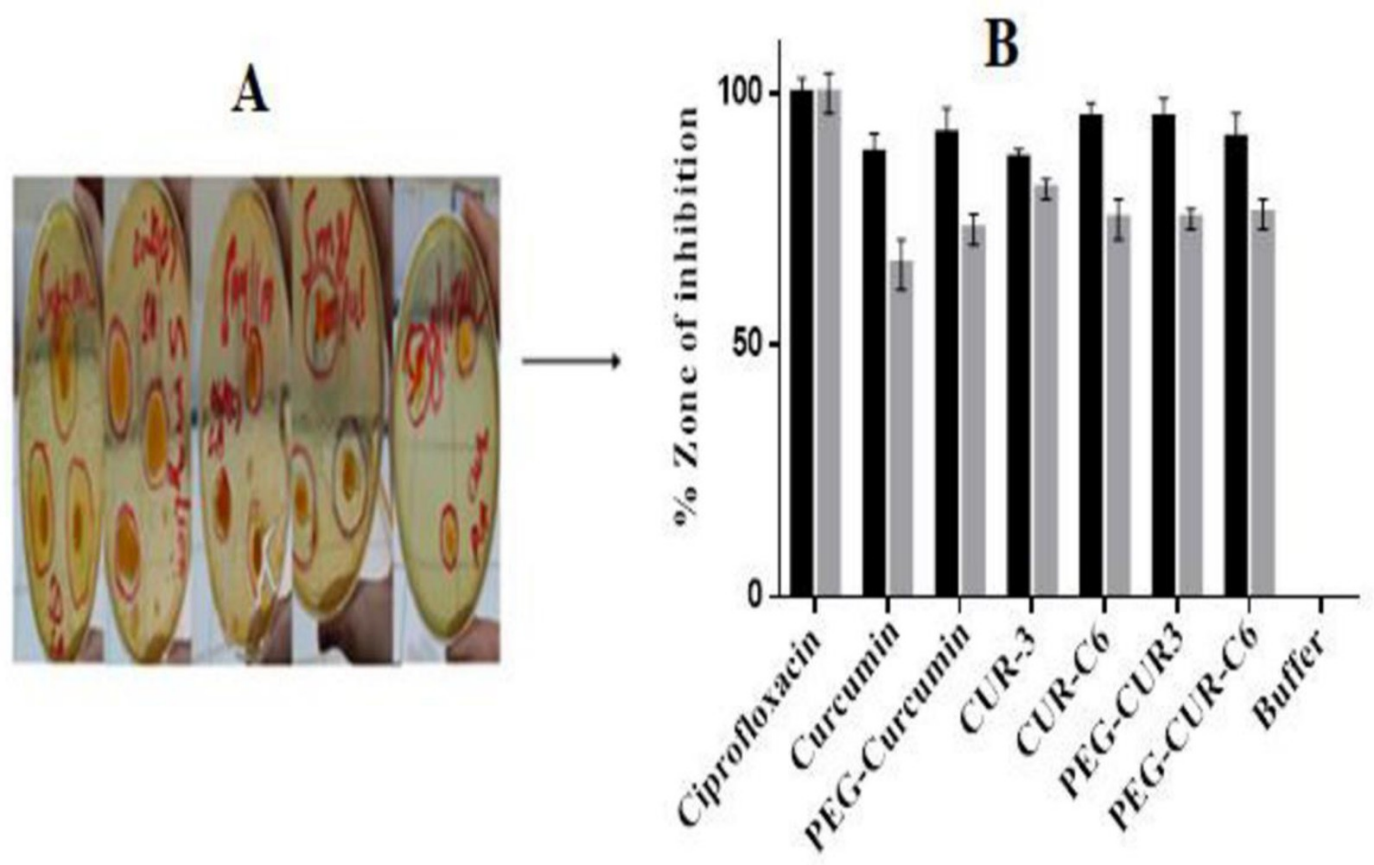
Antimicrobial activity of ciprofloxacin (standard), curcumin, PEG-curcumin, CUR-3, CUR-C6, PEG-CUR3, PEG-CUR-C6 ointments in various concentration shown in (A) against (■) *Staphylococcus aureus* (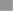) *Pseudomonas aeruginosa* and graph shown in (B). Test was performed in triplicate mean ± SD (n = 3).

The antimicrobial susceptibility analysis of curcumin, PEG-Curcumin, CUR-3, CUR-C6, PEG-CUR3, PEG-CUR-C6 was performed against gram negative bacteria *“S aureus”* and gram-positive bacteria *“P aeruginosa”* by measuring zone of inhibition. Maximum zone of inhibition (ZOI) against *S*. *aureus* was measured 27mm with 3mg/100μl of PEG-CUR3 and PEG-CUR-C6 and 25mm with 3mg/100μl against *P*.*aeruginosa*. Minimum ZOI was measured 20mm with 1mg/100μl concentration against *S*. *aureus* and 19mm measured with 1mg/100μl concentration against *P*. *aeruginosa*. Percentage ZOI of Ciprofloxacin (5μg/100μl) was taken 100% as standard while ZOI of curcumin, PEG-Curcumin, CUR-3, CUR-C6, PEG-CUR3, PEG-CUR-C6 against *S*. *aureus* was 88, 92, 87, 95, 95, 91% and against *P*.*aeruginosa* was 66, 73, 81,75, 75, 76% respectively. The maximum percentage of anti-microbial effect of PEG-CUR-C6 was due more soluble PEG-CUR complex in ophthalmic ointment and permeation enhancer cremophore. In another study A.K Alzomor *et al*., [31] also reported the similar behavior of CUR (Fig 8).

## 4. Conclusion

The analysis of several clinical and preclinical investigations shows that curcumin was used as a therapeutic agent in the treatment of various eye diseases such as corneal bacterial infections, glaucoma, cataract, and diabetic retinopathy. Improved curcumin solubility and corneal penetration was observed with the help of PEG-CUR complex and addition of cremophore in ointment formulations. Prepared optimized ointment formulation PEG-CUR-C6 was found to have good solubility, corneal permeation, low toxicity and maximum ZOI against *S*. *aureus* and *P*. *aeruginosa* showing enhanced antibacterial effect. Results showed that optimized PEG-CUR oleaginous base ointment with penetration enhancer cremophore was considered a promising tool to treat the bacterial infections of cornea and vitreous humour also improved permeation of cornea.

## Supporting information

S1 Graphical abstract (DOCX)

S1 Dataset (DOCX)

## References

[1] Üstündağ Okur N, Çağlar EŞ, Siafaka PI. Novel ocular drug delivery systems: An update on microemulsions. Journal of Ocular Pharmacology and Therapeutics. 2020;36(6):342–54. doi: 10.1089/jop.2019.0135 32255728

[2] Sai N, Dong X, Huang P, You L, Yang C, Liu Y, et al. A novel gel-forming solution based on PEG-DSPE/Solutol HS 15 mixed micelles and gellan gum for ophthalmic delivery of curcumin. Molecules. 2020;25(1):81. 10.3390/molecules25010081 PMC6983186 31878332

[3] Ohno M, Nishida A, Sugitani Y, Nishino K, Inatomi O, Sugimoto M, et al. Nanoparticle curcumin ameliorates experimental colitis via modulation of gut microbiota and induction of regulatory T cells. PLoS One. 2017;12(10):e0185999. doi: 10.1371/journal.pone.0185999 28985227 PMC5630155

[4] Radomska-Leśniewska DM, Osiecka-Iwan A, Hyc A, Góźdź A, Dąbrowska AM, Skopiński P. Therapeutic potential of curcumin in eye diseases. Central-European journal of immunology. 2019;44(2):181. doi: 10.5114/ceji.2019.87070 31530988 PMC6745545

[5] Davis BM, Pahlitzsch M, Guo L, Balendra S, Shah P, Ravindran N, et al. Topical curcumin nanocarriers are neuroprotective in eye disease. Scientific reports. 2018;8(1):1–13. doi: 10.1038/s41598-017-17765-5 30038334 PMC6056418

[6] Salma H, Melha YM, Sonia L, Hamza H, Salim N. Efficient Prediction of In Vitro Piroxicam Release and Diffusion From Topical Films Based on Biopolymers Using Deep Learning Models and Generative Adversarial Networks. Journal of pharmaceutical sciences. 2021. doi: 10.1016/j.xphs.2021.01.032 33548245

[7] Wang T-y, Chen J-x. Effects of curcumin on vessel formation insight into the pro-and antiangiogenesis of curcumin. Evidence-Based Complementary and Alternative Medicine. 2019;2019. doi: 10.1155/2019/1390795 31320911 PMC6607718

[8] Hoshikawa A, Nagira M, Tane M, Fukushige K, Tagami T, Ozeki T. Preparation of curcumin-containing α-, β-, and γ-cyclodextrin/polyethyleneglycol-conjugated gold multifunctional nanoparticles and their in vitro cytotoxic effects on A549 cells. Biological and Pharmaceutical Bulletin. 2018;41(6):908–14. doi: 10.1248/bpb.b18-00010 29863079

[9] Xu X, Al-Ghabeish M, Krishnaiah YS, Rahman Z, Khan MA. Kinetics of drug release from ointments: role of transient-boundary layer. International journal of pharmaceutics. 2015;494(1):31–9. doi: 10.1016/j.ijpharm.2015.07.077 26241753

[10] Nguyen TN-G, Tran PH-L, Van Vo T, Van Tran T, Tran TT-D, editors. Dissolution enhancement of curcumin by solid dispersion with polyethylene glycol 6000 and hydroxypropyl methylcellulose. 5th International Conference on Biomedical Engineering in Vietnam; 2015: Springer.

[11] Kumar VS, Rijo J, Sabitha M. Guargum and Eudragit® coated curcumin liquid solid tablets for colon specific drug delivery. International journal of biological macromolecules. 2018;110:318–27. doi: 10.1016/j.ijbiomac.2018.01.082 29378277

[12] Bhagurkar AM, Angamuthu M, Patil H, Tiwari RV, Maurya A, Hashemnejad SM, et al. Development of an ointment formulation using hot-melt extrusion technology. AAPS PharmSciTech. 2016;17(1):158–66. doi: 10.1208/s12249-015-0453-3 26628438 PMC4766123

[13] Patil P, Parit S, Waifalkar P, Patil S, Dongale T, Sahoo SC, et al. pH triggered curcumin release and antioxidant activity of curcumin loaded γ-Fe2O3 magnetic nanoparticles. Materials Letters. 2018;223:178–81.

[14] Yousef SA, Mohammed YH, Namjoshi S, Grice JE, Benson HA, Sakran W, et al. Mechanistic evaluation of enhanced curcumin delivery through human skin in vitro from optimised nanoemulsion formulations fabricated with different penetration enhancers. Pharmaceutics. 2019;11(12):639. doi: 10.3390/pharmaceutics11120639 31805660 PMC6956259

[15] Polat HK, Pehlivan SB, Özkul C, Çalamak S, Öztürk N, Aytekin E, et al. Development of besifloxacin HCl loaded nanofibrous ocular inserts for the treatment of bacterial keratitis: In vitro, ex vivo and in vivo evaluation. International journal of pharmaceutics. 2020;585:119552. doi: 10.1016/j.ijpharm.2020.119552 32569814

[16] Severino P, Andreani T, Jäger A, Chaud MV, Santana MHA, Silva AM, et al. Solid lipid nanoparticles for hydrophilic biotech drugs: Optimization and cell viability studies (Caco-2 & HEPG-2 cell lines). European journal of medicinal chemistry. 2014;81:28–34. doi: 10.1016/j.ejmech.2014.04.084 24819957

[17] Narayani SS, Saravanan S, Ravindran J, Ramasamy M, Chitra J. In vitro anticancer activity of fucoidan extracted from Sargassum cinereum against Caco-2 cells. International journal of biological macromolecules. 2019;138:618–28. doi: 10.1016/j.ijbiomac.2019.07.127 31344415

[18] Liu F, Gao S, Yang Y, Zhao X, Fan Y, Ma W, et al. Curcumin induced autophagy anticancer effects on human lung adenocarcinoma cell line A549. Oncology letters. 2017;14(3):2775–82. doi: 10.3892/ol.2017.6565 28928819 PMC5588543

[19] Sandle T. Antibiotics and preservatives. Pharmaceutical Microbiology: Essentials for Quality Assurance and Quality Control; Sandle, T, Ed. 2016:171–83.

[20] Fouad SA, Malaak FA, El-Nabarawi MA, Abu Zeid K, Ghoneim AM. Preparation of solid dispersion systems for enhanced dissolution of poorly water soluble diacerein: In-vitro evaluation, optimization and physiologically based pharmacokinetic modeling. PLoS One. 2021;16(1):e0245482. doi: 10.1371/journal.pone.0245482 33471832 PMC7816977

[21] Wang R, Han J, Jiang A, Huang R, Fu T, Wang L, et al. Involvement of metabolism-permeability in enhancing the oral bioavailability of curcumin in excipient-free solid dispersions co-formed with piperine. International journal of pharmaceutics. 2019;561:9–18. doi: 10.1016/j.ijpharm.2019.02.027 30817985

[22] Yin X, Cao X, Li J, Cheng X, Cheng G, Zou M, et al. A novel surfactant-free O/O paclitaxel ointment for the topical treatment of psoriasis. AAPS PharmSciTech. 2019;20(5):1–16. doi: 10.1208/s12249-019-1413-0 31165303

[23] Aboali FA, Habib DA, Elbedaiwy HM, Farid RM. Curcumin-loaded proniosomal gel as a biofreindly alternative for treatment of ocular inflammation: In-vitro and in-vivo assessment. International Journal of Pharmaceutics. 2020;589:119835. doi: 10.1016/j.ijpharm.2020.119835 32890654

[24] Zhang L, Pan X, Xu L, Zhang L, Huang H. Mitochondria-targeted curcumin loaded CTPP–PEG–PCL self-assembled micelles for improving liver fibrosis therapy. RSC Advances. 2021;11(10):5348–60. 10.1039/d0ra09589c PMC8694685 35423083

[25] Singh P, Gupta V. Curcumin loaded deformable drug carrier for the disease of posterior segment of eye: diabetic retinopathy. 2021.

[26] Gangurde AB, Kundaikar HS, Javeer SD, Jaiswar DR, Degani MS, Amin PD. Enhanced solubility and dissolution of curcumin by a hydrophilic polymer solid dispersion and its insilico molecular modeling studies. Journal of Drug Delivery Science and Technology. 2015;29:226–37.

[27] Sun S, Du X, Fu M, Khan AR, Ji J, Liu W, et al. Galactosamine-modified PEG-PLA/TPGS micelles for the oral delivery of curcumin. International Journal of Pharmaceutics. 2021;595:120227. doi: 10.1016/j.ijpharm.2021.120227 33484915

[28] Faralli A, Shekarforoush E, Ajalloueian F, Mendes AC, Chronakis IS. In vitro permeability enhancement of curcumin across Caco-2 cells monolayers using electrospun xanthan-chitosan nanofibers. Carbohydrate polymers. 2019;206:38–47. doi: 10.1016/j.carbpol.2018.10.073 30553335

[29] Moiseev RV, Morrison PW, Steele F, Khutoryanskiy VV. Penetration enhancers in ocular drug delivery. Pharmaceutics. 2019;11(7):321. doi: 10.3390/pharmaceutics11070321 31324063 PMC6681039

[30] Bucolo C, Drago F, Maisto R, Romano GL, D’Agata V, Maugeri G, et al. Curcumin prevents high glucose damage in retinal pigment epithelial cells through ERK1/2-mediated activation of the Nrf2/HO-1 pathway. Journal of cellular physiology. 2019;234(10):17295–304. doi: 10.1002/jcp.28347 30770549

[31] Alzomor AK, Noman NM, Al-Qubati A, Al-Shawafi A, Al-Serry A, Al-Zedaar S. Development of Anti-bacterial Ointment from Two Extracts of Curcuma longa L. and Aloe vera L. Journal of Pharmaceutical Research International. 2017:1–13.

