# Supplementary material for: Improved solubility and corneal permeation of PEGylated curcumin complex used for the treatment of ophthalmic bacterial infections
Source: PLoS One. 2022 Apr 7;17(4):e0258355. doi: 10.1371/journal.pone.0258355 (PMC8989353; doi:10.1371/journal.pone.0258355)
Supplement: S1 Graphical abstract — (DOCX) [file pone.0258355.s001.docx]

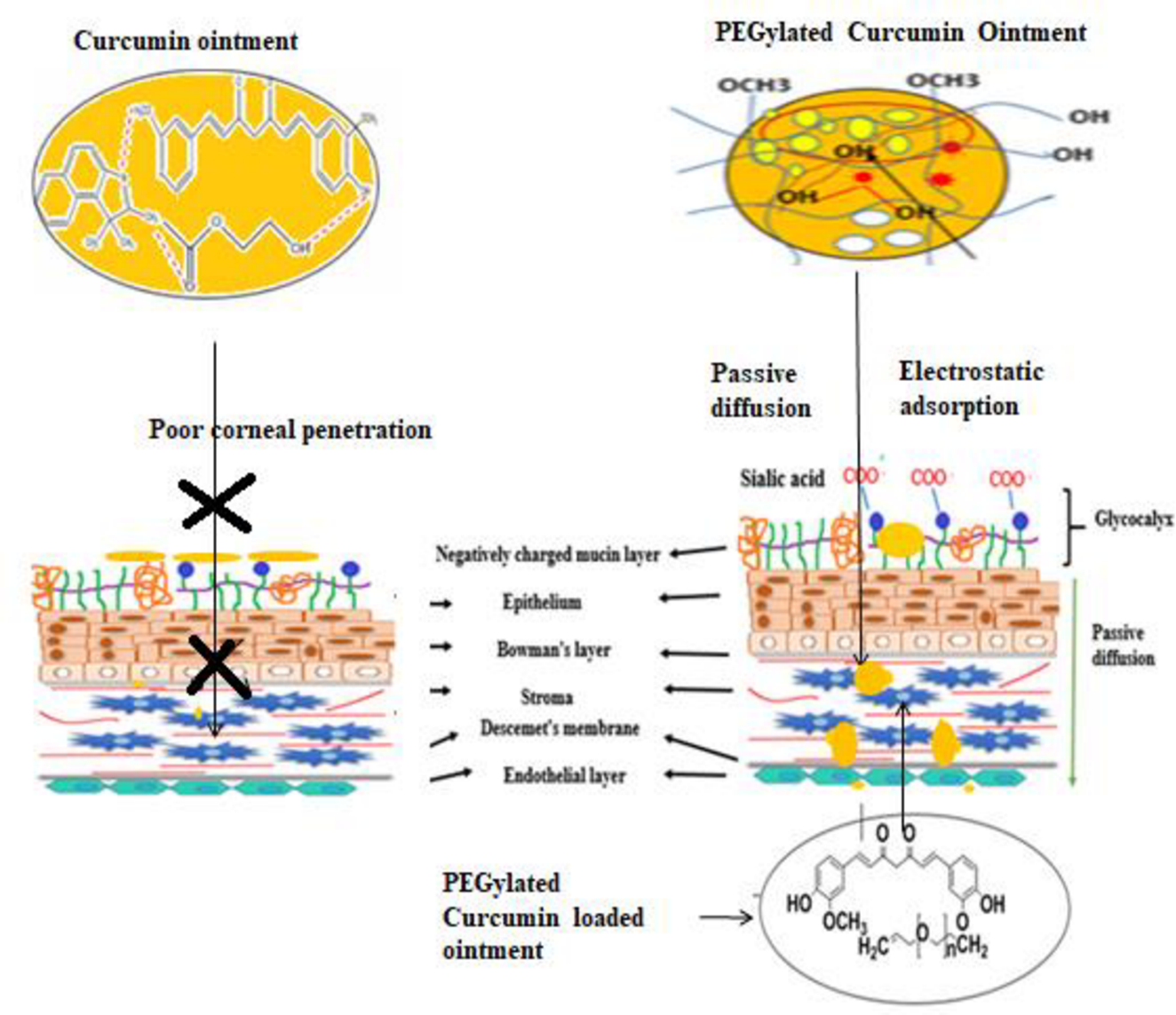


Graphical abstract showing comparison between mechanisms of permeation of CUR ointment and PEG-CUR ointment containing oleaginous bases and cremophore
