# Supplementary material for: Improved solubility and corneal permeation of PEGylated curcumin complex used for the treatment of ophthalmic bacterial infections
Source: PLoS One. 2022 Apr 7;17(4):e0258355. doi: 10.1371/journal.pone.0258355 (PMC8989353; doi:10.1371/journal.pone.0258355)
Supplement: S1 Dataset — (DOCX) [file pone.0258355.s002.docx]

**Minimal Data set of required figures**

The minimal data set of figures 2,5,6,7 and 8 is written as fellows while figures 1, 3, and 4 are chemical scheme, central composite rotatable design (CCRD) 3D model images and FTIR spectra respectively.

| **Fig #** |  | **Mean** | **S.D** | **S.E** | **# Samples** |
| --- | --- | --- | --- | --- | --- |
| **Figure 2** | Molar conc. of curcumin (M) | 0.0031  0.0063  0.0333  0.0128  0.0114  0.009  0.0076  0.0051 | 0.0002  0.0009  0.035  0.0005  0.0002  0.0003  0.0004  0.0002 | 0.004  0.011  0.192  0.004  0.002  0.003  0.004  0.003 | 8 |
| **Figure 5** | | | | | |
| **%Absorption of CUR-3 from 0.25 to 4 hours** | | | | | |
|  | Time (hr)  0.25  0.5  1  2  3  4 | 1.79  2.12  2.38  2.52  2.14  2.56 | 1.22  1.09  2.82  2.86  2.89  2.79 | 0.91  0.74  1.82  1.80  1.97  1.74 | 6 |
| **%Absorption of PEG-CUR3 from 0.25 to 4 hours** | | | | | |
|  | Time (hr)  0.25  0.5  1  2  3  4 | 2.47  2.53  3.14  3.78  3.81  3.83 | 3.2  2.4  1.56  2.54  2.39  4.41 | 2.03  1.50  0.88  1.30  1.22  2.25 | 6 |
| **%Absorption of PEG-CUR-C6 from 0.25 to 4 hours** | | | | | |
|  | Time (hr)  0.25  0.5  1  2  3  4 | 2.44  2.49  3.27  3.79  4.6  5.83 | 2.78  2.81  2.57  2.69  2.36  3.95 | 1.77  1.78  1.42  1.38  1.10  1.63 | 6 |
| **Figure 6** | | | | | |
| **%Diffusion of CUR-3 from 1 to 6 hours** | | | | | |
|  | Time (hr)  1  2  3  4  5  6 | 6  10  7.5  6.7  7.1  7.4 | 3.44  1.62  5.83  5.5  2.89  2.79 | 1.40  0.51  2.12  2.12  1.08  1.02 | 6 |
| **%Diffusion of** **PEG-CUR3 from 1 to 6 hours** | | | | | |
|  | Time (hr)  1  2  3  4  5  6 | 11.6  12.5  13  14.1  14.7  15.6 | 7.24  6.95  3.47  5.82  5.94  4.71 | 2.12  1.96  0.96  1.55  1.54  1.19 | 6 |
| **%Diffusion of PEG-CUR-C6 from 1 to 6 hours** | | | | | |
|  | Time (hr)  1  2  3  4  5  6 | 13.4  14.5  15.2  16  17.8  18.3 | 8.21  7.83  5.77  7.35  5.4  6.12 | 2.24  2.05  1.47  1.83  1.27  1.43 | 6 |
| **Figure 7** | | | | | |
| **Cell viability studies of different formulations of curcumin at 3 hours** | | | | | |
|  | Positive control (MEM)  Negative control (Triton) %  Curcumin 0.5%  Curcumin 1%  CUR-C6 0.5%  CUR-C6 1%  PEG-Curcumin 0.5%  PEG-Curcumin 1%  PEG-CUR-C6 0.5%  PEG-CUR-C6 1% | 100  4.43  70.33  143  131.6  125.3  70.7  145.3  136.6  107.3 | 5.90  1.22  8.52  3.00  6.04  9.50  4.50  7.51  5.04  10.50 | 0.59  0.57  1.01  0.25  0.52  0.84  0.53  0.62  0.43  1.01 | 10 |
| **Cell viability studies of different formulations of curcumin at 24 hours** | | | | | |
|  | Positive control (MEM)  Negative control (Triton) %  Curcumin 0.5%  Curcumin 1%  CUR-C6 0.5%  CUR-C6 1%  PEG-Curcumin 0.5%  PEG-Curcumin 1%  PEG-CUR-C6 0.5%  PEG-CUR-C6 1% | 100  3.16  90.33  85  102  81.3  82.66  76.66  59  69.33 | 5.90  0.29  6.51  7.0  10.6  8.72  11.03  5.50  8.60  4.72 | 0.59  0.16  0.68  0.75  1.04  0.96  1.21  0.62  1.11  0.56 | 10 |
| **Figure 8** | | | | | |
| **%Zone of inhibition against *Staphylococcus aureus*** | | | | | |
|  | Ciprofloxacin  Curcumin  PEG-Curcumin  CUR-3  CUR-C6  PEG-CUR3  PEG-CUR-C6 | 100  88  92  87  95  95  91 | 3  4  5  2  3  4  5 | 0.3  0.42  0.52  0.21  0.30  0.41  0.52 | 7 |
| **%Zone of inhibition against *Pseudomonas aeruginosa*** | | | | | |
|  | Ciprofloxacin  Curcumin  PEG-Curcumin  CUR-3  CUR-C6  PEG-CUR3  PEG-CUR-C6 | 100  66  73  81  75  75  76 | 4  5  3  2  4  2  3 | 0.4  0.61  0.35  0.22  0.46  0.23  0.34 | 7 |
